# Non-surgical management of tubal ectopic pregnancy: A systematic review and meta-analysis

**DOI:** 10.1097/MD.0000000000027851

**Published:** 2021-12-17

**Authors:** Chao Xiao, Qingquan Shi, Qijun Cheng, Jianli Xu

**Affiliations:** a Department of Obstetrics and Gynecology, Zigong First People's Hospital, Zigong, China; b Center of Reproductive Medicine, West China Second University Hospital, Sichuan University, Chengdu, Sichuan Province, China; c Key Laboratory of Birth Defects and Related Diseases of Women and Children (Sichuan University), Ministry of Education, Chengdu, Sichuan Province, China; d Department of Obstetrics and Gynecology, West China Second University Hospital, Sichuan University, Chengdu, Sichuan Province, China.

**Keywords:** ectopic pregnancy, methotrexate, mifepristone, non-surgical management

## Abstract

**Background:**

Ectopic pregnancy (EP) is a common cause of acute abdominal pain in the field of gynecology. Because the majority of women with EP are hemodynamically stable, non-surgical therapy is a viable option. The goal of this study was to determine the most effective non-surgical therapy for hemodynamically stable EP.

**Methods:**

We performed a systematic review and meta-analysis. We searched PubMed, LILACS, SciELO, CINAHL, Embase, and the Cochrane library in May 2020, with no starting date restrictions.Studies were restricted to randomized controlled trials, which were included if the target population contained women with tubal EP and the intervention was non-surgical management. The primary outcome measure was treatment success defined by a decrease in serum hCG to a level ranging from five mIU/mL to 50 mIU/mL. Secondary outcome measures were side effects, time needed to treat, number of injections and operative rate.

**Results:**

We conducted a meta-analysis of 15 studies that included 1573 women who were diagnosed with EP and managed non-surgically. There was no significant difference in treatment success in the matched groups; however, single-dose MTX was associated with fewer side effects than multiple-dose (relative risk 0.48, 95% confidence interval 0.28–0.80, *P* = .006) and two-dose therapies (relative risk 0.74, 95% confidence interval 0.55–1.00, *P* = .05).

**Conclusions:**

We highly recommend that single-dose MTX without mifepristone be used first-line in patients who require conservative therapy due to the inherent negative effects of mifepristone. An EP woman with a low -hCG level that is falling or plateauing should receive expectant treatment to reduce adverse effects.

## 1 Introduction

Ectopic pregnancy (EP) is a common cause of acute abdominal pain in gynecology. The incidence of EP is 2% to 3%.^[1,2]^ Tubal pregnancy is the most common form of EP (composing up to 90% of cases) and is also the leading cause of death in early pregnancy.^[3]^ Typical clinical manifestations of ectopic pregnancy include amenorrhea, abdominal pain, vaginal bleeding, syncope and shock. If a woman suffers from EP, her future fertility can be jeopardized. Fortunately, EP is being diagnosed earlier in recent years because of advancements in imaging technology and protocols to screen women at risk.^[4,5]^ Rapid immunoassay of serum human chorionic gonadotrophin (β-hCG) has been widely used to clinically diagnose pregnancy. If these women are asymptomatic, however, more than 90% of EPs can be detected by high-resolution transvaginal ultrasonography. If the β-hCG level is 3500 mIU/mL or higher, the sensitivity and specificity of transvaginal ultrasonography in diagnosing EP ranges from 87.0% to 99.0% and 94.0% to 99.9%, respectively.^[6]^

Currently, the treatment options for women with EP are surgical, medical, and expectant management. Surgical methods are salpingectomy and salpingostomy by laparoscopy or laparotomy. Medical treatments include methotrexate (MTX), mifepristone and traditional Chinese medicine (TCM)^[4,27]^. Surgery is suitable for EPs with cardiac complications or hemodynamic instability^[3]^. However, most EPs that are diagnosed early are stable, which makes non-surgical therapeutic methods possible. Additionally, the inherent drawbacks of surgical treatment are anesthesia complications, secondary injuries and blood loss. In contrast, non-surgical management can avoid these problems.

MTX is the mainstay of medical management of EP. The drug acts as an anti-folic acid, anti-tumor agent and has been identified as an inhibitor of the JAK/STAT pathway by many independent threads of evidence.^[7]^ The most common regimens are single-dose (i.e., MTX 50 mg/m^2^ intramuscular injection), two doses (i.e., 50 mg/m^2^ injected on days 1 and 4), and multiple doses (i.e., 1 mg/kg intramuscular injection on days 1, 3, 5, ± 7).

Mifepristone is an anabolic steroid with a structure that is similar to norethindrone. Because its affinity to the progesterone receptor is five times higher than that of progesterone^[8,9]^, it can competitively bind to the progesterone receptor in the decidua and inhibit the activity of progesterone, resulting in degeneration of villi tissue, atrophy and necrosis of the decidual tissue, eventually leading to embryo death.

TCM has a unique advantage in the treatment of hemodynamically stable EP, as this approach can activate blood circulation, remove blood stasis and kill the embryo.^[10]^ It can promote phagocytosis and improve the absorption of plasma protein by peritoneal lymphatic vessels.

Expectant management of EP can also be performed because early EP is a self-limiting disease resulting in tubal absorption.

Currently, there are no standards for hemodynamically stable EP regarding medication, dose, dosage regimen, duration, etc. Therefore, we analyzed non-surgical treatments to determine an appropriate therapeutic method for patients with stable EP.

## 2 Materials and methods

### 2.1 Search strategy

Studies were identified by searching PubMed, LILACS, SciELO, CINAHL, Embase, and the Cochrane library in May 2020, with no starting date restrictions. Combinations of the following keywords were used to identify the studies: “mifepristone,” “methotrexate,” “ectopic pregnancy,” “tubal pregnancy,” “Traditional Chinese medicine,” and “expectant management.” No filters were applied for language or location. Two investigators evaluated all identified trials and separately assessed the methodological quality of the included studies. Any discrepancies were solved by mutual discussion. Ethical approval and consent of patient were not applicable for our meta-analysis, because we just included published literature.

### 2.2 Study selection and data extraction

Studies were restricted to RCTs, which were included if the target population contained women with tubal EP and the intervention was non-surgical management. The primary outcome measure was treatment success defined by a decrease in serum hCG to a level ranging from 5 mIU/mL to 50 mIU/mL. Secondary outcome measures were side effects, time needed to treat, number of injections and operative rate. We defined drug therapy by type (MTX, mifepristone or TCM) and route of administration (intramuscular injection or oral administration). Two reviewers (Chao Xiao and Qingquan Shi) independently judged all abstracts filtered by the search strategies. Full texts of all eligible studies were obtained to assess whether these studies met the predefined inclusion criteria. Differences in opinion were registered and resolved by consensus with all authors.

### 2.3 Outcome measures

The primary outcome was treatment success. The secondary outcomes were side effects, time needed to treat, number of injections and operative rate.

### 2.4 Study quality assessment

For RCTs, we used the risk bias assessment according to the criteria in the Cochrane Handbook for Systematic Reviews of Interventions.

### 2.5 Statistical analysis

Statistical analysis was conducted according to the guidelines for reviewers of the Cochrane Menstrual Disorders and Subfertility Group. The treatment result was expressed as relative risk (RR) with a 95% confidence interval (CI) in each study. If sufficient data were available, a summary statistic for each outcome was calculated using a fixed effect model. Statistical heterogeneity between the results of studies was examined by inspecting the distribution of the data points on graphs and the overlap of CIs and by checking the *I*^2^ statistic. A value of ≥50% was treated as substantial heterogeneity. In the case of statistical heterogeneity, the original trials were checked for differences in patient characteristics. Review Manager (RevMan5.3, Cochrane Collaboration, Oxford, UK) was used for the statistical analysis, and a *P* < .05 was considered significant.

## 3 Results

### 3.1 Study characteristics

**Figure 1:**
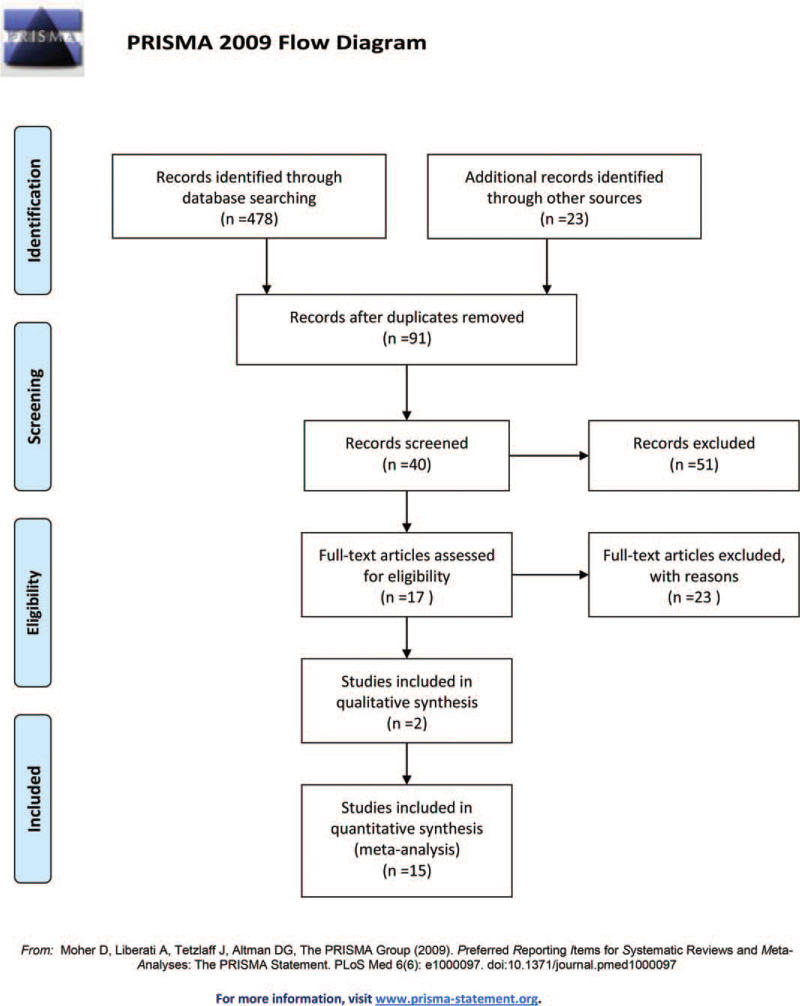
Study selection flowchart of the meta-analysis.

Ninety-one reports were identified using the search strategy after duplicates were excluded. The remaining studies were screened by title and abstract. The study identification and selection process are presented as a flowchart in Fig. 1. The characteristics of the included studies are shown in Table 1.

**Table 1:** Characteristics of included studies.

							Outcome				
NO.	Author	Study/Location	Participants	Random method	Intervention	Control	O1	O2	O3	O4	O5
1	M.R.Gazvani et al.(1998)	RCT/UK	TM<4cm;Confirmed laparoscopically	Computer-generated	Single dose (25)	SDCM (25)	Success rate	second injection	Operative treatment	Nausea	
2	Patrick Rozenberg et al.(2003)	RCT/France	HS;β-hCG<1500mIU/ml and its increase less 50% over 48 h;β-hCG>1500mIU/ml and no intrauterine sac	computer-generated	Single dose (97)	SDCM (113)	Success rate	second injection	Operative treatment	Nausea	Pelvic pain
3	Zhuo-hua et al.(2004)	RCT/China	HS;TM<5cm;β-hCG<2000mIU/ml	No detail	Single dose (102)	SDCM (80)	Success rate	HCG level	Diameter of the pelvic mass		
4	Chad K.et al.(2005)	RCT/USA	clinical diagnosis	NO detail	Single dose (22)	Multidose (29)	Success rate	Side effects	Surgery rate	TTR	
5	Alleyassin.et al.(2006)	RCT/Iran	HS;TM<3.5cm;β-hCG<1800 mIU/ml and Plateauing levels or <50% increase over 48 hours 3.stable hemodynamic;β-hCG≥1800 mIU/ml and no intrauterine sac;	computer-generated	Single dose (54)	Multidose (54)	Success rate	Side effects			
6	EMINE SEDA.et al.(2010)	RCT/Turkey	HS;TM<3.5cm;β-hCG increase less 50% over 48 h	computer-generated	Single dose (62)	Multidose (58)	Success rate	Side effects	TTR		Pregnancy rate
7	Tabatabaii.et al.(2012)	RCT/Iran	HS;TM<4cm;β-HCG <15000 mIU/mL;Absence of gestational cardiac activity	computer-generated	Single dose (35)	Multidose (35)	Success rate	Side effects	Repeat dose	Surgery rate	Surgery rate
8	Korhonen et al.(1996)	RCT/Finland	TM<4cm;hCG<5000IU/L;No fetal cardiac	table of random numbers	MTX orally (30)	Expectant (30)	Success rate	Surgery rate			
9	N.M. van Mello.et al.(2012)	RCT/Netherlands	HS;HCG<1500 mIU/ml;Ectopic Sac visible on TVUS	web-based mrandomization	Single dose (39)	Placebo (32)	Success rate	Side effects	Repeat dose	Surgery rate	Surgery rate
10	Priscila.et al.(2014)	RCT/Brazil	HS;TM<5.0cm;HCG<2000 mIU/ml	no detail	Single dose (10)	Saline (13)	Success rate	TTR			
11	JURKOVIC.et al.(2016)	RCT/UK	HS;HCG<1500 mIU/ml;ultrasound diagnosis of EP	computer-generated	Single dose (35)	Saline (36)	Success rate	Surgery rate	Intra-abdominal bleeding		
12	Hossam O. Hamed.et al.(2012)	RCT/Egypt	HS;TM<4cm;β-HCG<15000 mIU/mL	computer-generated	Single dose (78)	Two dose (79)	Success rate	Side effects	Repeat dose	Surgery rate	LOFU
13	Najmieh Saadati.et al.(2015)	RCT/Iran	HS;β-HCG<15000 mIU/mL	Block randomization	Single dose (69)	Two dose (79)	Success rate	Side effects	Repeat dose	Surgery rate	LOFU
14	Song.et al.(2015)	RCT/South Korea	HS;TM<4cm;β-HCG<15000mIU/mL	Randomly permuted blocks	Single dose (46)	Two dose (46)	Success rate	Side effects	Repeat dose	Surgery rate	LOFU
15	Hend S.et al.(2016)	RCT/Egypt	HS;TM<4cm;β-HCG<6000 mIU/mL	Computer-generated	Single dose (80)	Two dose (80)	Success rate	Side effects	LOFU		

HS = hemodynamically stable, LOFU = length of follow-up, SDCM = single dose combined mifepristone, TM = tubal mass, TTR = time to resolution of hCG values.

The 15 included studies were RCTs; Four^[9,11–13]^ did not give details of the randomization method. Overall, we reviewed 1573 cases of women diagnosed with EP in our meta-analysis. All patients were hemodynamically stable with adnexal masses < 4 cm. The upper limit of serum hCG was 15000 IU/L, and gestational cardiac activity was absent.

All trials were published as full papers, and 1 study was published as a conference abstract only.^[11]^ Three trials were performed in Turkey, Iran and the USA; two in the UK, Egypt and France, and the remaining studies were in Korea, Finland, Brazil, New Zealand, and the Netherlands.

The outcome measures (i.e., treatment success and side effects) are presented in Forest plots. No heterogeneity (*I*^2^) was found in any subgroup except for the follow-up group of Single dose versus two doses (*I*^2^ = 99%).

### 3.2 Quality assessment

Eleven of the 15 RCTs reported the randomization details, and 10 studies reported adequate blinding. All studies reported the outcome data and described selective reporting. The included RCTs were of high quality. The main characteristics of the 15 included trials are presented in Tables 2 and 3.

**Table 2:**
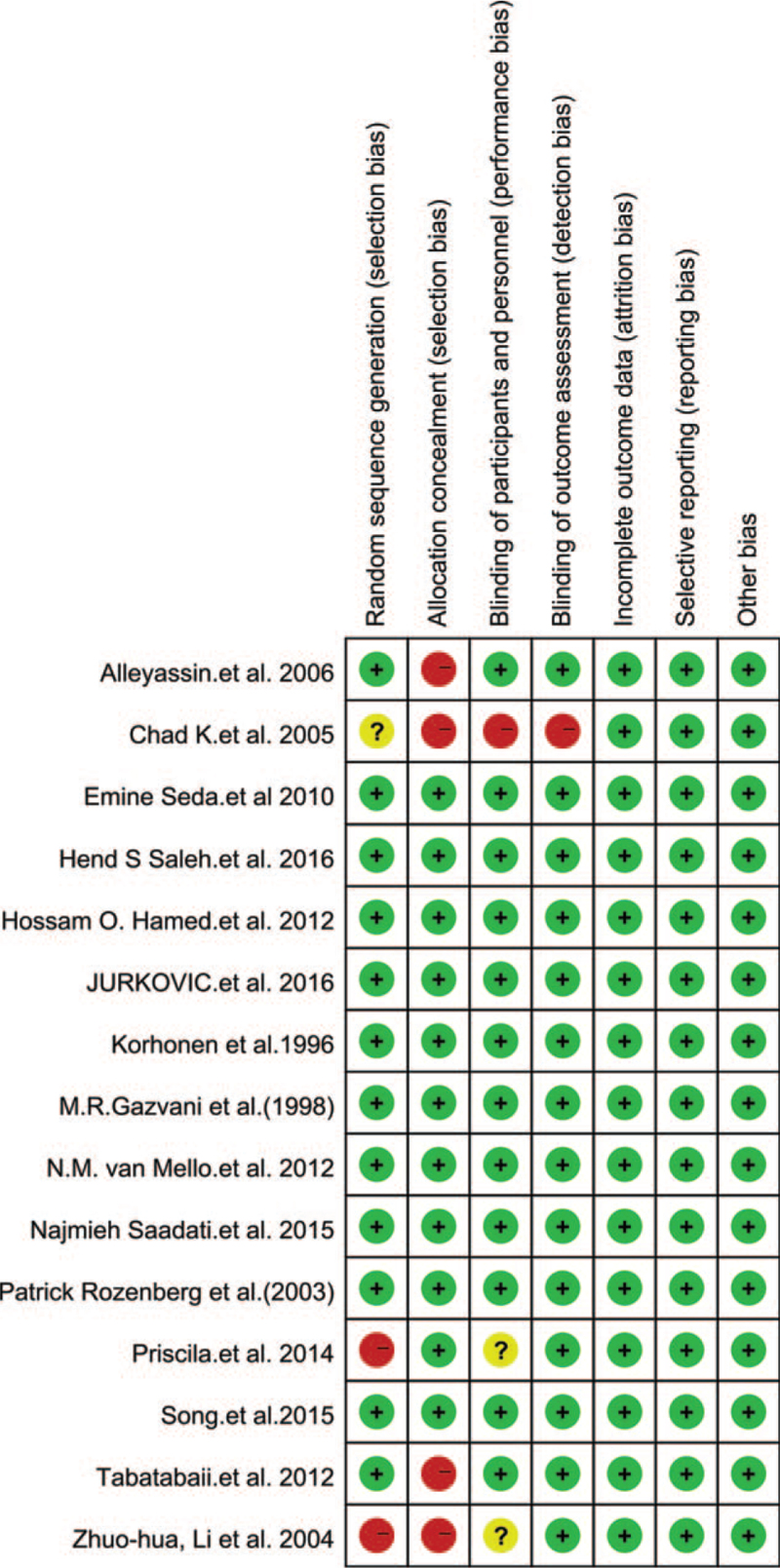
Risk of bias summary using cocharane risk assessment tools.

**Table 3:**
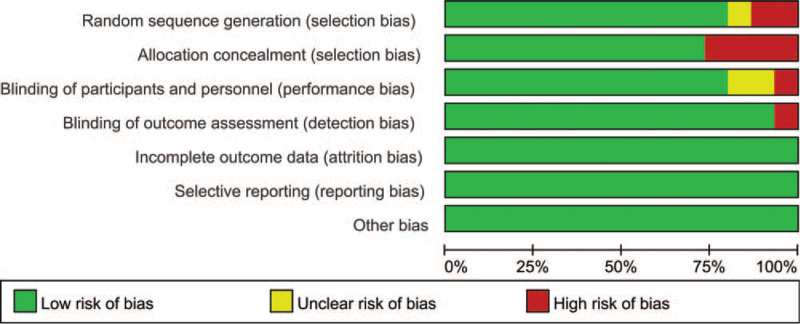
Rish of bias graph.

### 3.3 Single-dose MTX versus single-dose MTX combined with mifepristone

**Figure 2:**
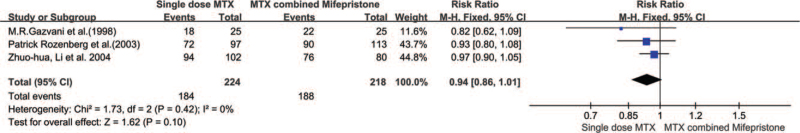
Forest plots of success rate.

EPs were given an injection of 50 mg/m^2^ MTX alone or in combination with 600 mg of oral mifepristone. In three studies that included 442 women, the variance in treatment success between single-dose MTX and single-dose MTX combined with mifepristone was significant (Chi^2^ = 1.73, df = 2 (*P* = .42), *I*^2^ = 0%). We then used a fixed effect model to estimate combined RR. Mifepristone plus a single dose of MTX did not result in a higher success rate than MTX injection alone (RR 0.89, 95% CI 0.86–1.01, *P* = .10) (Fig. 2).

**Figure 3:**
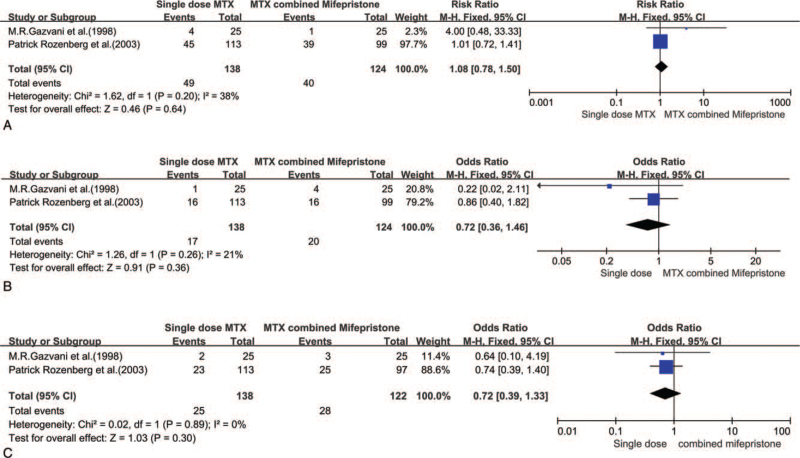
A: Forest plots of side effects, B: Forest plots of second injection. C: Forest plots of operative rate.

The variance in the subgroups (side effects, second injection and operative rate) was not significant (*I*^2^ ranged from 0% to 38%), so we used a fixed effect model to estimate the combined RR. The results of these three subgroups were not significant (*P* ranged from .3 to .64) (Fig. 3A-3C).

### 3.4 Single versus multiple doses of MTX

**Figure 4:**
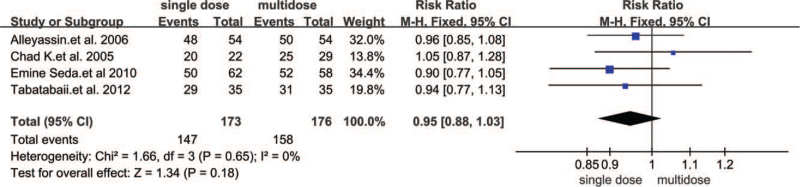
Forest plots of success rate.

Three trials reported that adnexal masses were < 3.5 cm,^[12–14]^ and 1 study^[15]^ stated that the adnexal masses were < 4 cm. The highest β-hCG cut-off reported in the study by Tabatabaii^[15]^ and Alleyassin^[13]^ was less than 15,000 mIU/mL. A fixed effect model was used to calculate the combined RR because heterogeneity was 0%. The combined results of 6 trials involving 992 tubal EPs showed that treatment success associated with a single dose of systemic MTX (50 mg/m^2^ or 1 mg/kg i.m.) was not significantly different from that of multiple doses of MTX (RR 0.95, 95% CI 0.88–1.03, *P* = .18) (Fig. 4).

**Figure 5:**
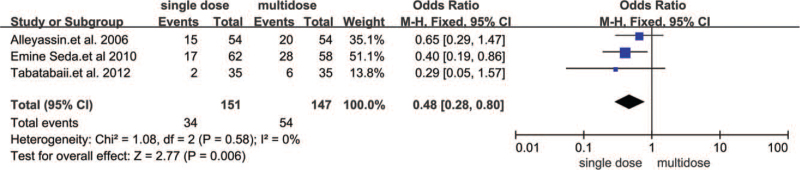
Forest plots of side effects.

Three trials including 198 women reported the side effects of MTX. Side effects were significantly lower than those who received multiple doses of MTX (RR 0.48, 95% CI 0.28–0.80, *P* = .006) (Fig. 5).

### 3.5 Single-dose versus Placebo (expectant management)

One double-blind placebo controlled study^[16]^ included 60 patients who received systemic MTX (2.5 mg/day MTX orally for five days); 1 study^[17]^ involved single-dose injection of MTX (1 mg/kg i.m. with a maximum of 100 mg); and two trials^[18,19]^ involved a single dose of 50 mg/m^2^. Serum hCG concentrations in the studies were defined as below 2000 IU/L, except 1 study^[16]^ used a level less than 5000 IU/L.

**Figure 6:**
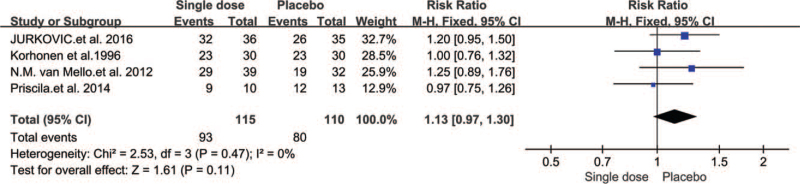
Forest plots of success rate.

We included four trials involving 225 stable EPs. The results showed that single-dose MTX was not associated with a higher success rate than the expectant method (RR 1.13, 95% CI 0.97–1.30, *P* = .11) (Fig. 6).

**Figure 7:**
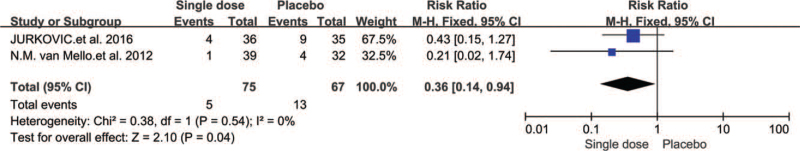
Forest plots of operative rate.

Only two studies^[17,19]^ reported patients who required surgery. All patients were diagnosed with EP by ultrasound and had an hCG < 1500 mIU/ml. The study by van Mello et al.^[17]^ included pregnancy of unknown location, and hCG levels were less than 2000 mIU/ml. The results showed that EP patients undergoing expectant treatment had a higher incidence of surgery (RR 0.36, 95% CI 0.14–0.94, *P* = .04) (Fig. 7).

### 3.6 Single dose versus 2 doses

Six studies of 557 EPs were included in this subgroup. The inclusion criteria of these trials were the following: upper limit of serum hCG (6000–15000 mIU/mL), absent fetal heartbeat, hemodynamic stability, and adnexal mass size was < 4 cm. Mean serum hCG concentrations in women treated with MTX varied between 493 and 14891 mIU/mL. The single-dose MTX treatment was 50 mg/m^2^, and the two-dose MTX regimen involved 50 mg/m^2^ given on days one and four.

**Figure 8:**
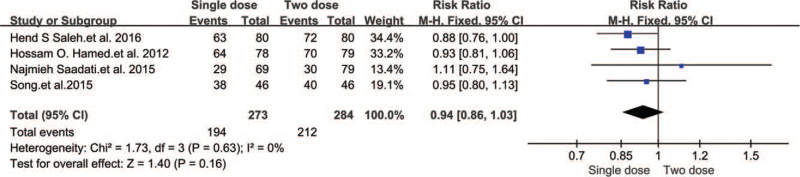
Forest plots of success rate.

Our analysis of initial intervention success showed that there was no significant difference between the two regimens (RR 0.94, 95% CI 0.86–1.03, *P* = .16) (Fig. 8).

**Figure 9:**
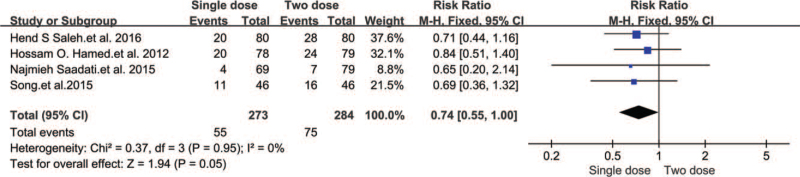
Forest plots of side effects.

The most common side effects were abdominal pain and nausea and/or vomiting. There were 557 EPs distributed across four trials, and our meta-analysis results showed that two-dose MTX had 1.94 times higher side effects than the single dose regimen (RR 0.74, 95% CI 0.55–1.00, *P* = .05) (Fig. 9).

**Figure 10:**
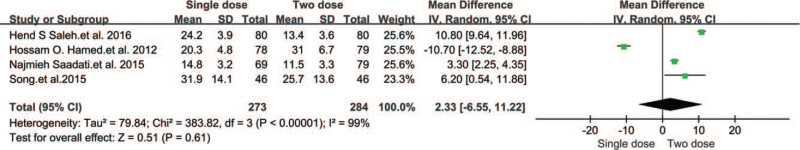
Forest plots of follow-up period.

The follow-up hCG level ranged from 5 mIU/ml to 15 mIU/ml across various studies, except for the study by Saleh et al^[20]^ in which they used 200 mIU/ml as the level of treatment success. Because the heterogeneity was 99%, we chose a random effect model to calculate the overall effect. Our results revealed that the length of follow-up was the same in the various groups (MD 2.33, 95% CI 6.55–11.22, *P* = .61) (Fig. 10).

## 4 Discussion

This systematic review has thoroughly investigated various non-surgical therapeutic strategies for EPs. In the analysis of trials of single-dose MTX compared to single-dose MTX combined with mifepristone, there was no difference in initial treatment success, side effects, number of injection and operative rate. This finding may relate to the low concentration of serum hCG. Spontaneous resolution occurred in 77% of cases if the median baseline hCG level was low.^[16]^ There is only one study^[21]^ included using a progesterone level cut-off of 10 nmol/L, and those EPs with a higher level of progesterone had resulted in a better resolution rate (*P* = .01). The other three trials did not analyze progesterone levels, so we were unable to perform a meta-analysis of this data point. A luteolytic effect may be key to mifepristone's therapeutic efficacy,^[22,23]^ so we suggest that future studies include progesterone level as a criterion.

Our meta-analysis revealed that treatment success did not significantly differ between multiple-dose and single-dose protocols or between two-dose and single-dose regimens. A similar result had been reported in a cohort trial^[12]^ in which success rates between multiple-dose and single-dose methotrexate therapy were comparable (95% and 90%, respectively; *P* = .18). Unfortunately, we were unable to conduct a subgroup analysis of hCG level because of the variability in hCG level cut-offs in the trials. Only Hamed et al^[24]^ reported a subgroup analysis of hCG level ranging from 3600 to 5500 mIU/ml, in which those who received two doses had a 5.8-fold higher success rate than those who received single-dose treatment (odds ratio = 0.58, 1.29–26.2, *P* = .03). Two trials^[14,25]^ that compared a single-dose with a two-dose regimen reported similar treatment success in the subgroup analyses of hCG level. This result is in agreement with previous meta-analyses.^[23]^ In contrast, the side effects associated with multiple doses and two doses were 2.77-fold and 1.94-fold higher than those of single-dose MTX, respectively. The lack of significant differences in success rate between the three systemic MTX regimens may relate to various factors. First, the initial single dose was much higher than that of multiple doses, and high-dose MTX may disrupt trophoblastic proliferation. Second, the overall total doses differed. Third, leucovorin, which was used in the multiple-dose group for reducing MTX efficacy, is a fully reduced folate-like compound that bypasses the methotrexate-inhibited dihydrofolate reductase enzyme and provides a pool of carbon donors for nucleic acid synthesis.^[26]^

Our meta-analysis revealed that single-dose MTX treatment did not significantly contribute to the success of expectant management of unruptured tubal EP. Success rates of expectant management ranged from 59% to 92% in two studies.^[15,17]^ According to van Mello et al,^[17]^ only 20% of patients had an EP diagnosed by ultrasound, while the remaining 80% of cases were pregnancies of unknown location, the majority of which were likely to be failed intrauterine pregnancies. All the participants had plateauing β-hCG levels, which many clinicians feel compelled to treat. These factors may explain the higher success rate in both arms of their trial compared with the other studies. The expectant group had a higher operative rate, because abdominal pain caused by tubal abortion or hematoma formation, and the gynecologist may feel inclined to perform surgery in fear of tubal rupture.

Because of the lack of RCTs in TCM, we only reviewed articles^[27,28]^ that used TCM as an adjuvant to MTX. The authors both concluded that TCM supported treatment success and a decrease in hCG level, as well as shortened the time to hCG resolution and decreased the recurrence of EP. We strongly recommend that future studies conduct RCTs to investigate the efficacy of TCM and use TCM as an adjuvant in EP treatment.

## 5 Limitations

The limitations of our meta-analysis are as follows. (1) The small number of RCTs and their small sample sizes led to insufficient statistical power, which affects the stability of the results. (2) The comparison of single-dose MTX and single-dose MTX combined with mifepristone did not consider progesterone level, so we suggest that future studies include this information. (3) The medical management of EP should take more detail subgroup by HCG level, the HCG ranged from 6000 to 15000 mIU/mL, so suggested to take the different cut-off as the inclusion criteria. (4) Because of the few RCTs investigating TCM, we were unable to conduct a meta-analysis of TCM research. RCTs of TCM should be conducted in the future.

## 6 Conclusion

First, the treatment success of all non-surgical treatments for EP did not differ, but systemic MTX was associated with more side effects. Second, in terms of ipsilateral tubal obstruction, single-dose MTX appears to be more helpful to maintain tubal potency than multiple-dose.^[29]^ Last, single-dose MTX is convenient not only for the patient but also for the physician and is associated with better fertility outcomes.^[15]^ So a single dose of MTX without mifepristone should be first-line for an EP patient who needs conservative management. If a patient with an EP has a low β-hCG level that is decreasing or plateauing, an expectant strategy should be implemented to mitigate side effects.

## Author contributions

**Conceptualization:** Chao Xiao.

**Data collection:** Chao xiao, Qijun Cheng.

**Formal analysis:** Chao xiao.

**Investigation:** Chao xiao, Qijun Cheng.

**Methodology:** Qingquan Shi.

**Project administration:** Chao xiao, Qijun Cheng.

**Resources:** Chao xiao, Qingquan Shi.

**Software:** Chao xiao, Qingquan Shi.

**Supervision:** Jianli Xu, Qingquan Shi.

**Validation:** Jianli Xu.

**Visualization:** Chao xiao, Qijun Cheng.

**Writing – review & editing:** Chao xiao, Qingquan Shi.
